# Consumer Mobile Apps for Potential Drug-Drug Interaction Check: Systematic Review and Content Analysis Using the Mobile App Rating Scale (MARS)

**DOI:** 10.2196/mhealth.8613

**Published:** 2018-03-28

**Authors:** Ben YB Kim, Anis Sharafoddini, Nam Tran, Emily Y Wen, Joon Lee

**Affiliations:** ^1^ Health Data Science Lab School of Public Health and Health Systems University of Waterloo Waterloo, ON Canada

**Keywords:** drug interactions, telemedicine, mobile applications, smartphone, consumer health informatics, consumer health information

## Abstract

**Background:**

General consumers can now easily access drug information and quickly check for potential drug-drug interactions (PDDIs) through mobile health (mHealth) apps. With aging population in Canada, more people have chronic diseases and comorbidities leading to increasing numbers of medications. The use of mHealth apps for checking PDDIs can be helpful in ensuring patient safety and empowerment.

**Objective:**

The aim of this study was to review the characteristics and quality of publicly available mHealth apps that check for PDDIs.

**Methods:**

Apple App Store and Google Play were searched to identify apps with PDDI functionality. The apps’ general and feature characteristics were extracted. The Mobile App Rating Scale (MARS) was used to assess the quality.

**Results:**

A total of 23 apps were included for the review—12 from Apple App Store and 11 from Google Play. Only 5 of these were paid apps, with an average price of $7.19 CAD. The mean MARS score was 3.23 out of 5 (interquartile range 1.34). The mean MARS scores for the apps from Google Play and Apple App Store were not statistically different (*P*=.84). The information dimension was associated with the highest score (3.63), whereas the engagement dimension resulted in the lowest score (2.75). The total number of features per app, average rating, and price were significantly associated with the total MARS score.

**Conclusions:**

Some apps provided accurate and comprehensive information about potential adverse drug effects from PDDIs. Given the potentially severe consequences of incorrect drug information, there is a need for oversight to eliminate low quality and potentially harmful apps. Because managing PDDIs is complex in the absence of complete information, secondary features such as medication reminder, refill reminder, medication history tracking, and pill identification could help enhance the effectiveness of PDDI apps.

## Introduction

Potential drug-drug interactions (PDDI) have been a prevalent source of preventable problems that can occur in any age group and increase costs to the health care systems [1]. A PDDI occurs when an individual is prescribed two drugs that are known to interact. An occurrence of drug-drug interaction (DDI) is defined as a clinical alteration of the exposure or response to a drug as a result of coadministration. DDIs can be clinically relevant when the result of the interaction warrants the attention of health care professionals (HCPs). When the outcome of the DDI is harmful, it is referred to as an adverse drug reaction (ADR) [2]. DDIs have a profound impact on the safety of patients, and it has been found to be involved in 26% of all ADR-related hospital admissions [3]. Furthermore, in the United States, emergency visits because of ADR cost in average US $3704 per patient [4,5], demonstrating a huge economical impact.

Most PDDIs are preventable, but it remains a significant problem to patients and the health care system [3,6]. It has been observed that physicians are not always aware of clinically significant drug interactions [7,8] and may underestimate the effects of PDDIs [9]. Other factors such as high workload in pharmacy could also lead to higher risk of PDDIs for patients [10,11]. DDIs have also been identified as a significant portion of the overall ADRs resulting in hospitalization among older adults [12].

One possible solution that has been proposed is to use a decision support system to detect and avoid PDDIs [7,9]. With the rise of smartphones and mobile apps, decision support systems for PDDIs are now within the reach of consumers and patients and no longer exclusive to HCPs. This is an opportunity that can engage and empower patients by providing necessary tools to detect, avoid, and report ADR events stemming from DDIs [13-17]. The potential benefit for older adults with polypharmacy—the use of multiple medications—is deemed greater because of multiple prescribing providers involved in the care, which is a substantial risk factor for medication errors and ADR events [18].

Mobile health (mHealth) apps with PDDI decision support are not subject to the Food and Drug Administration regulation [19], and this may pose a substantial threat to the safety of consumers and patients. To our knowledge, the quantity, features, characteristics, or efficacy of the available PDDI mHealth apps on the market have never been systematically assessed. Therefore, understanding the characteristics of these mHealth apps is important in planning future interventions or policies aiming at patient-centered care and patient safety. This study systematically reviewed and assessed PDDI decision support mHealth apps available in Canada through the Google Play Store (Google Inc, Canada) and Apple’s App Store (Apple Inc, Canada) using the Mobile App Rating Scale (MARS) [20].

## Methods

### Systematic Review Design

This systematic review adhered to the Preferred Reporting Items for Systematic Reviews and Meta-Analyses systematic review protocol [21] as closely as possible, but it deviated in few instances because of the characteristics of mHealth app databases, which differ from scholarly reference databases for published articles. To ensure the review process is transparent and replicable, the detailed descriptions of each step are provided below.

### App Search Strategy

Our review aimed to search apps that were publicly available to Canadians in English. Two most popular mobile app databases, Apple’s App Store and the Google Play Store, which account for over 80.0% of mobile apps market in 2016 [22], were searched in this study.

This study developed a keyword search procedure to identify potentially eligible apps (Textbox 1). First, the searcher was instructed to log out from the Google account on a browser to prevent any personalized search results. Apple’s App Store and the Google Play Store were searched with the search terms related to drug interactions. The search terms were specifically developed to be in all lower case letters and in quotations for consistent and comprehensive search results. As operating systems and apps are updated routinely, searches on both stores were conducted on the same day in December 2016. Additionally, the searches were performed on a designated set of devices and the same network to obtain consistent search results and avoid deviations by personalized search results [23]. Search results were extracted and saved in a spreadsheet for the next stage of app selection.

### App Selection

Following the search of the two databases, for each search term, all the identified apps were screened in two stages. First, the reviewers verified the eligibility of the apps against the inclusion criteria by reading the apps’ descriptions available in the app stores. This study included apps that claim to check for PDDIs in their description, published in English, and last updated in 2016 or later. Apps were excluded if they targeted nongeneral consumers, passively informed users of PDDIs (does not allow pair-wise or combinational interaction check), checked for drug interactions for pets and animals, and specific to a particular disease or drug class. After screening the results for each search term, the selected app names were aggregated. If an app was listed in both stores, this study considered them separately and examined both versions to capture potentially varying features and user reviews. Second, the authors downloaded and installed the remaining apps from the first step to verify their eligibility one more time. Apps that failed to launch after three attempts on the test devices were excluded. All Apple test devices ran iPhone operating system (iOS, Apple Inc) 10, and all Android test devices ran Android 6.0.

### Data Collection Process

A set of general information about the apps were extracted following previous app review studies [24,25]. General app information provides contextual information such as availability, affordability, and user satisfaction level. A set of secondary features that can further empower end users beyond the PDDI check feature was identified from literature review [24,26-28].

**Search strategy with an example for Google Play Store.**

Preparing your device for the search:

- Connected to the University of Waterloo network
- Log out from Google in your browser

Search procedure:

- Search the following terms in the respective store
- Search terms must be in quotation (eg, “drug interaction”)
- All search terms should be entered in lower case letters

Search terms (number of hits)

1. drug interaction (66)
2. drugs interaction (8)
3. drug interactions (193)
4. drugs interactions (16)
5. drug-interaction (66)
6. pill interaction (3)
7. pills interaction (0)
8. pill interactions (3)
9. pills interactions (0)
10. pill-interaction (3)
11. medication interaction (10)
12. medications interaction (0)
13. medication interactions (192)
14. medications interactions (0)
15. medication-interaction (10)

In summary, the two extracted sets of information were as follows: (1) general information about the apps: last updated date, price, and user rating and (2) other relevant secondary features that the apps offered:

- Medication management related features: reminder to take medication, reminder to refill medication, medication history tracking, pill identification, searching medication using generic or brand names, and access to medication database
- Security and privacy related features: password protection for user data and multiple user support
- Data sharing and social media: sharing user data with a third party
- Clinician and technical support: customer support

Multimedia Appendix 1 presents the secondary features extracted and examined for each app.

### Critical Appraisal of the Apps (Quality Assessment)

The MARS, a 23-item, expert-based rating scale with a purpose of assessing the quality of mHealth apps, was used to critically and systematically evaluate the quality of the mHealth apps [20] (See Multimedia Appendix 2 for a detailed MARS score for all included apps). Each question from MARS used a 5-point scale (1=inadequate, 2=poor, 3=acceptable, 4=good, and 5=excellent). This expert scale consists of multiple dimensions that assess different quality aspects of apps, including end-user engagement, features, aesthetics, content quality, and subjective quality [20]. This expert rating scale has been increasingly adopted in recent years for evaluating mHealth apps such as mindfulness [29], weight loss [25,30], smoking cessation [30], self-care [31], online well-being [32], and medication adherence [24]. A previous study has shown high internal consistencies in the total score and subscales, as well as strong interrater reliability (IRR) [20]. Moreover, use of a standardized assessment scale such as MARS for evaluating mHealth apps has been recommended by various researchers [33-35]. The popularity of MARS led to the further development of an Italian version [36] and an end-user version for nonresearchers [37].

The last dimension of MARS is app subjective quality, which takes the subjective opinions of the reviewers. To ensure the quality assessment process is as consistent and objective as possible, the subjective quality dimension was omitted from this review. A previous study that employed MARS as an objective method to assess quality also excluded the subjective quality dimension [25]. Instead, relevant information was captured from the app databases, including the price and app ratings.

Before rating the apps, each rater read and familiarized themselves with the MARS protocol. A group discussion was followed to achieve a consensus on the rating criteria, and the first app was rated as a group. The need for an objective example of PDDIs arose for the MARS questions (#15 and #16) that assess comprehensiveness and accuracy of the content and information. On the basis of a careful review of the literature [38,39], we developed a list of PDDIs with 20 true positive and six false positive examples (Multimedia Appendix 3). The percentage of correctly identified and described PDDIs was scaled to a range from 1 to 5 for questions #15 and #16. No previous studies have reported on the details of how the accuracy and comprehensiveness of app content were assessed.

Two raters assessed each app individually. Weighted kappa, Krippendorff alpha, and intraclass correlation (ICC) were used to estimate IRR for MARS tool. The kappa value was assessed by putting quadratic weights for differing values. The ICC coefficient was calculated with a two-way random model and for agreement level. The weighted kappa, Krippendorff alpha, and ICC were calculated per dimension and for all apps.

### Statistical Analyses

Each dimension in MARS was analyzed using the mean value as recommended by the developers [20]. The difference in app quality between the two app stores was analyzed by *t* tests. The relationships among four dimensions of the MARS score—MARS total score, price, average rating, and number of features—were examined by the Spearman correlation. A significance level of .05 was used in this study. All analyses were performed in R version 3.3.2 (R Foundation for Statistical Computing, Vienna, Austria).

## Results

### Systematic Search Results

**Figure 1:**
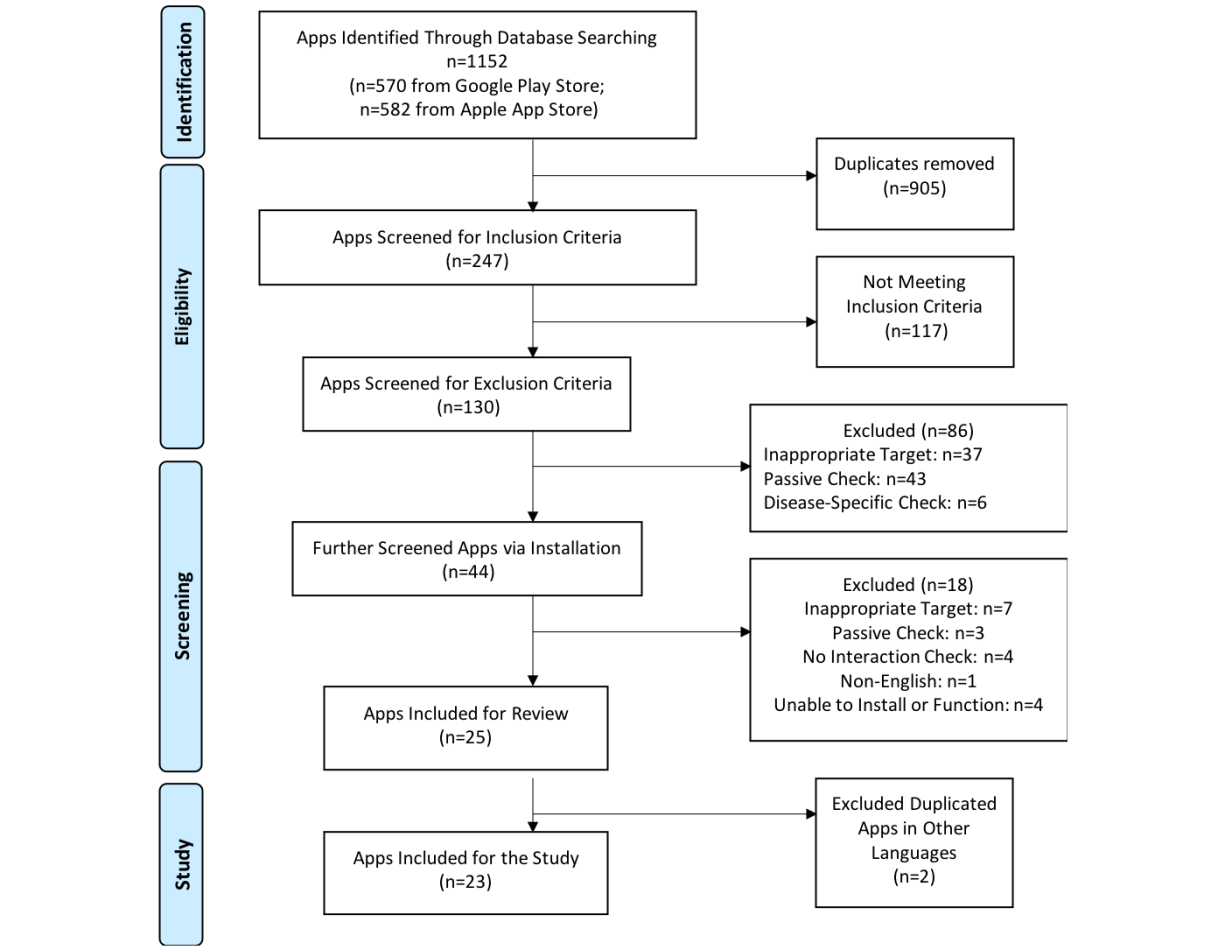
App selection process.

The app store search was conducted in December 2016. This study identified 570 apps from Google Play and 582 apps from Apple App Store (Figure 1). After removing duplicates in each database, the authors reviewed the descriptions of 247 apps against the inclusion and exclusion criteria (Figure 1). Apps found to be eligible based on their descriptions (n=44) were installed for another round of review against the criteria (Figure 1). Review was initiated for 25 apps, but the authors excluded two additional apps identified as duplicates in multi-language versions, leaving a total of 23 apps for this study (Figure 1).

### General Information

Twenty-three apps—12 from Apple App Store and 11 from Google Play—were developed by 15 developers. Seven apps were listed in both stores. Table 1 summarizes the general information of the reviewed apps and the mean MARS scores.

There were five paid apps, three from Apple App Store and two from Google Play, with an average price of $7.19 CAD. The average prices of paid apps were $7.32 CAD and $6.99 CAD for Apple’s App Store and the Google Play Store, respectively. Four apps, two apps from each store, “Drug Interactions” and “Prescription Checker” by the same developer, were functionally identical but listed at two different prices: $10.99 CAD and $6.99 CAD in Apple’s App Store and $9.33 CAD and $4.65 CAD in the Google Play Store, respectively.

The last updated dates for the Android apps were from April 2016 to December 2016, whereas the iOS apps ranged from July to December 2016.

The average rating for the apps from the Google Play Store was 3.82, with a minimum of 2.1 and a maximum of 4.8 (interquartile range, IQR 0.85). On the other hand, the apps from Apple’s App Store averaged 4.5 based on the two apps with valid user ratings.

### App Features

**Figure 2:**
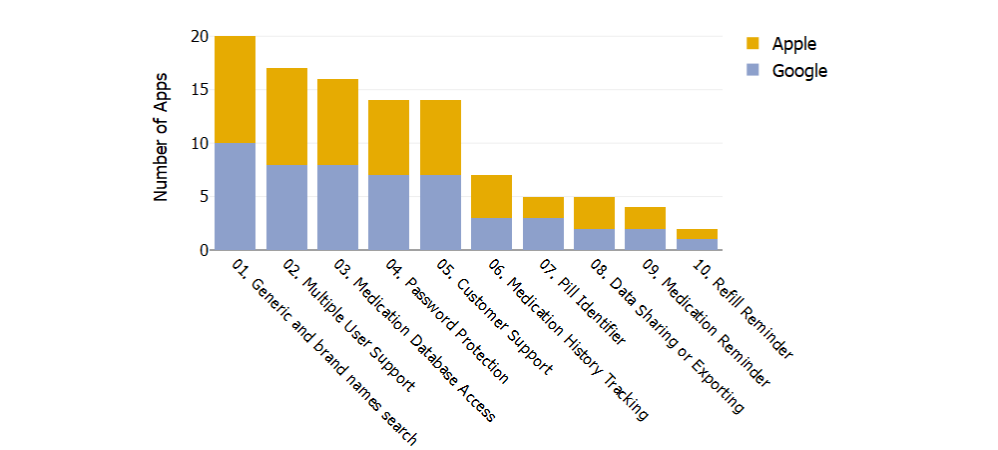
The number of apps that contain the secondary features listed on the x-axis.

Secondary features, features other than PDDI check, were extracted and examined for each app. On average, they had 3.67 features with a minimum of zero for “DrugChecker— Interactions (Lite)” and a maximum of eight for GenieMD in both stores (IQR 3). The overall number of apps per secondary feature is shown in Figure 2. Medication refill reminder was among the least incorporated features (2/23). The option to search medications with their generic and brand name (20/23), multiple user support (17/23), access to the app’s medication database (16/23), password protection (14/23), and customer support (14/23) were the most common features.

**Table 1:** General information about the eligible apps, developer, tested version, cost, average rating, and mean Mobile App Rating Scale (MARS) score. iOS: iPhone operating system. NA: not available.

App number and name		Platform	Developer	Tested app version	Cost ($ CAD)	Average rating (out of 5)	Mean MARS score (out of 5)
1	Drug center—pediatric oncall	iOS	Pediatric Oncall	3	Free	NA	3.15
2	Drug interactions	iOS	Pierre Chaillet	1.5.3	10.99	NA	2.29
3	DrugChecker—Interactions (Lite)	iOS	SYSTEM YOSHII	1.2.1	Free	NA	2.00
4	Drugs.com Medication Guide	iOS	Drugsite Trust	2.7.24	Free	4	4.06
5	GenieMD	iOS	GenieMD	7.4	Free	5	3.92
6	MyRxProfile	iOS	MyRxProfile	1.0.2	Free	NA	3.02
7	PharmaGuide	iOS	Asif Baig	1.0.5	Free	NA	1.94
8	Pharmazam	iOS	Pharmazam	2	Free	NA	2.94
9	Pharmacist Pro—Drug Interactions Checker	iOS	Yury Dubovoy	2	3.99	NA	3.60
10	PillSync Drug Facts Identifier	iOS	ScanIDme	1.2	Free	NA	3.23
11	Prescription Checker	iOS	Pierre Chaillet	1.1	6.99	NA	2.29
12	ZibdyHealth	iOS	Zibdy	1.5	Free	NA	2.29
13	Assist IE—Drug Interactions	Android	Infomed Mobile	1.0.41	Free	3.9	3.60
14	Assist UK—Drug Interactions	Android	Infomed Mobile	1.0.41	Free	4.0	3.60
15	CVS Caremark	Android	CVS Caremark	4.15	Free	3.5	4.10
16	Drug Center—Pediatric Oncall	Android	Pediatric Oncall	3.2	Free	4.4	3.33
17	Drug Interactions	Android	Pierre Chaillet	1.5.4	$9.33	2.7	2.29
18	Drugs.com Medication Guide	Android	Drugs.com	2.0.7.28	Free	4.3	4.06
19	Epocrates Plus	Android	Epocrates	17.1	Free	4.3	4.25
20	GenieMD	Android	GenieMD	5.9.9.54	Free	4.8	3.75
21	PillSync Drug Facts Identifier	Android	ScanIDme	4.3.0	Free	2.1	2.29
22	Prescription Checker	Android	Pierre Chaillet	1.5.4	$4.65	3.5	2.29
23	ZibdyHealth	Android	Zibdy	2.0	Free	4.5	3.60

**Figure 3:**
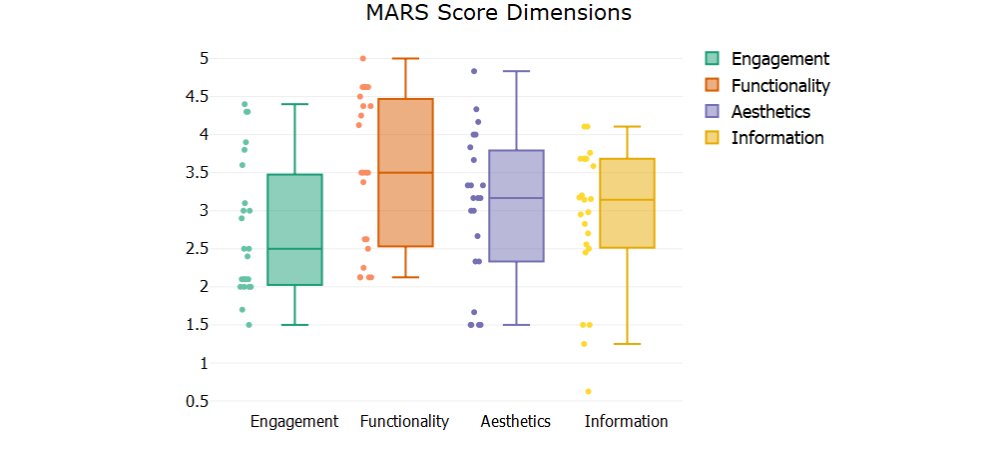
Mobile App Rating Scale (MARS) dimension scores. Each point represents the score for an individual app. The box plot shows the median, first, and third quartiles and minimum and maximum scores.

### Critical Appraisal of App Quality

#### Overall App Quality

The mean MARS score of the 23 apps was 3.05 (IQR 1.55), with a maximum of 4.40 for “Epocrates Plus” and a minimum of 1.87 for “PharmaGuide” (Table 1). The mean MARS score for the apps from Google Play and App Store were comparable at 3.25 and 2.86, respectively, with no statistical difference (*P*=.96). The IRR between two raters as assessed by the weighted kappa was .63 (95% CI 0.58-0.68), the ICC was .64 (95% CI 0.59-0.68), and the Krippendorff alpha was .63 (95% CI 0.58-0.66). Detailed IRR results are presented in Multimedia Appendix 4.

The mean scores of the four dimensions of MARS were examined to investigate the magnitude of the differences in quality in each dimension. Functionality dimension resulted in the highest mean score (3.52), whereas engagement dimension showed the lowest average score (2.75). The functionality dimension had the most variability (Figure 3).

### Relationships Between App Characteristics and Quality

General and functional characteristics of the 23 apps were examined for a correlation with the MARS score (Table 2). The general and functional characteristics, including average user rating and total number of features, were statistically significantly associated with the total MARS score (Table 2). Statistically significant associations were observed between the general and functional characteristics, including the total number of features and the price and average user rating (Table 2). Within the MARS dimensions, all were statistically significantly correlated with each other except for information dimension (Table 2).

**Table 2:** Correlations among total Mobile App Rating Scale (MARS) score, four MARS dimension scores, price, rating, and number of features.

Characteristics		MARS					Price	Average rating	Number of features
		Total	Engagement	Feature	Aesthetics	Information			
**MARS**									
	Total	1.00							
	Engagement	.87^a^	1.00						
	Feature	.72^a^	.49^b^	1.00					
	Aesthetics	.88^a^	.91^a^	.64^a^	1.00				
	Information	.43^b^	.30	−.08	.12	1.00			
Price		−.37	−.49^b^	−.47^b^	−.55^a^	.35	1.00		
Rating		.61^a^	.49^b^	.39	.41^b^	.43^b^	−.26	1.00	
Number of features		.47^b^	.70^a^	.06	.56^a^	.16	−.43^b^	.42^b^	1.00

^a^*P*<.01.

^b^*P*<.05.

## Discussion

### Principal Findings

In this app review study, a systematic search strategy was used to find PDDI apps. To our knowledge, this is the first systematic review on apps that offers decision support for PDDI checking. The 23 included apps were analyzed to extract general characteristics and functional characteristics, and their quality was assessed using MARS. Only five of the 23 apps (22%) were paid apps. This proportion of paid apps is consistent with other studies that systematically reviewed the Google Play Store and Apple’s App Store [24,25]. App price had statistically significant negative correlations with three of four MARS dimensions and number of features. This demonstrates that app quality is not always represented by the selling price. A plausible explanation for this counterintuitive and inverse relationship is that free apps may have been developed by companies and organizations with sufficient resources; hence, apps were developed to expand consumer reach, whereas individual developers who may have limited resources may rely on generating revenue from app sales, while the quality of app may not be as high as the apps developed by companies and organizations that can afford to hire a group of expert developers. Further research should investigate the relationship between the price of consumer mHealth apps and its quality, as well as its impact on consumer perception.

The primary features of the examined mHealth apps were providing drug information to users and checking for PDDIs. Despite this aim of these apps, a low average score in the information dimension was found based on MARS. This indicates that the PDDI check feature is of low quality, delivering inaccurate and potentially unsafe information about PDDIs. In particular, MARS questions #15 and #16, which assessed the accuracy and comprehensiveness, scored on average 2.9 and 2.4, respectively. This is alarming as only slightly more than half of 26 investigated PDDIs (58%, 3/5) have been identified by the apps. To worsen the problem, less than half of the correctly identified PDDIs (2.4 out of 5) have correctly described the interactions. Inability to detect PDDIs and providing incomplete and incorrect information is a significant threat to patient safety. It also diminishes mHealth app’s value as an avenue for patient empowerment. It must be noted that there was a large variability in the accuracy of PDDIs among the tested apps, where 48% (11/23) apps scored 4 or higher out of 5 for question #15, whereas 30% (7 / 23) apps scored less than 1 out of 5. This polarized quality of information found in mHealth apps further raises the question about the tools available for consumers to evaluate and select high quality apps. The average user rating was significantly correlated with the information dimension, and it indicates that the average user rating can potentially be an important tool for selecting mHealth apps. There are other resources available such as app clearinghouses that make recommendations for mHealth apps to consumers based on the results from systematically evaluating the usability, quality, accuracy, or evidence of the app and its content [40]. Examples of app clearinghouses include National Health Service Health App Library and iMedicalApps [40]. These app clearinghouses hold promise to enhance consumer safety of mHealth apps, but they have not been investigated against MARS or other validated tools that assess the quality of mHealth apps.

The low average MARS score for the engagement dimension can be partially explained by the primary purpose of the included apps. The investigated apps work as a reference to check for PDDIs, and these apps do not rely on user engagement to elicit behaviour change. On the other hand, other mHealth apps that focus on behaviour change tend to score higher in the engagement dimension, as the success of behaviour change may heavily depend on how successfully they engage the user [24].

Most MARS dimensions were correlated with each other except information. This is consistent with the findings from Bardus and colleagues who assessed weight management mHealth apps [25], where all dimensions but the information and engagement dimensions were significantly associated. A very strong correlation between the aesthetics and engagement dimensions can be explained by many user interface design, and usability studies that found attractive and appealing aesthetics lead to greater user engagement and perceived usability [41-43]. Interpreting the correlation between the total MARS score and each dimension’s score should take caution as the total MARS score is derived from the scores from all dimension of MARS. The number of features was strongly correlated with the engagement dimension but not with the features dimension that measures functionality, performance, and ease of use [20]. This result may represent the trade-off between ease of use and the complexity of an app that attempts to provide more features at the cost of performance. A similar relationship has been found in a previous website design and usability study [44].

### Secondary Features Offered to Consumers

Besides the PDDI check feature, maintaining medication adherence is a challenging problem in individuals taking medication, particularly for older adults [45,46] and those with chronic diseases [47]. Improving medication adherence can ensure the effectiveness of a treatment, thereby impacting maintaining health and managing chronic diseases [48]. There are many barriers for medication adherence, but forgetfulness has been reported as the most common cause, and much research has focused on overcoming this barrier [49,50]. A well-researched solution to overcoming forgetfulness is medication reminders and refill reminders [47,51]. Such reminders have increased patient medication adherence by encouraging timely refill and further demonstrated feasibility in cognitively impaired populations [47,51]. Therefore, these features can also be useful to individuals using PDDI apps. The usefulness of refill reminders has been acknowledged by the US government and made the refill reminder an exception to the Health Insurance Portability and Accountability Act [52]. Despite sufficient ground for implementing these features, only two apps featured a refill reminder (GenieMD in both stores), whereas five had a medication reminder.

Patients with comorbidities are usually cared for by a general physician and several specialists, which tends to lead to a heterogeneous list of medications [53,54]. The PDDI check feature can inspect for possible adverse effects, but this would be accurate only when the medication list is complete. Unfortunately, only 30% (7/23) of the reviewed apps had a feature to track medication history (Multimedia Appendix 1). Medication history tracking is also important in understanding PDDIs for drugs with long half-lives or over-the-counter drugs [55]. Therefore, mHealth apps that can track the history of medication can further prevent other drug complications. Such a feature can empower patients by enabling them to take charge of their medication list and minimizing PDDIs stemming from many HCPs with multiple prescriptions.

Every over-the-counter and prescription medication must have a unique appearance and imprint code for identification by the Food and Drug Administration [56]. Code imprint, size, color, and shape of the medication together permit identification of the product and manufacturer. However, using this identification system can be difficult for end users, and only 22% (5/23) of the apps had a feature to automatically identify pills from its physical attributes (Multimedia Appendix 1). Identification by drug name can also be difficult because of the discrepancies between generic and brand names. This review found that 87% (20/23) of the apps allow searching by both generic and brand names, and 70% (16/23) provide further drug information by allowing users to access a drug database. These features can help older adults who have developed polypharmacy to identify and distinguish drugs from one another, as a large number of medications and confusing names are often causes for medication error, even among trained clinicians [57].

Another issue that PDDI app users may be concerned about is data security as privacy is a major concern for collecting personal health information [58]. Overall, 61% (14/23) of the PDDI apps had password protection, and 74% (17/23) had support for multiple users on the same device (Figure 2). Given that smartphones and tablets can be protected with a password, an additional app-level password protection provides another level of security. Information and Privacy Commissioner of Ontario [59] and the Health Insurance Portability and Accountability Act state that password protection is required, but this may not be secure enough. Data encryption is recommended for added security. This is an area that can be greatly improved with a more stringent guideline and oversight by regulators and governments. Moreover, future research should investigate the level of data encryption presented in mHealth apps and its implication for consumers.

Our review also investigated availability of other features related to medication management in the apps. For instance, one study [60] has described data sharing via social networking sites as a potential communication platform to pharmaceutical companies to give feedback. Furthermore, social media can facilitate the interactions among patients, clinicians, researchers, and vendors [60]. The capabilities of data exporting, synthesized reporting for clinicians, and sharing on social media were found only in 22% (5/23) of the apps. In the context of mHealth apps that check for PDDIs, social media can provide a medium for consumers to interact with other drug users to share side effects and other relevant information. As Steele described [60], it may also help pharmaceutical companies interact with the users and gain insights into rare side effects, PDDIs, or high-risk subpopulations such as older adults.

Finally, in the event that information provided by the apps is not satisfactory, users should be able to get additional help. Of all, 61% of the apps (14/23) provided some level of customer support. Given the seriousness of potential ADRs that can be caused from the exposure to the PDDIs, an option to contact a clinician, preferably a pharmacist, would be ideal. It is worthwhile to note that no apps have provided contact information for reporting ADRs to local regulatory bodies. Providing a formal way to report potential ADRs to regulatory bodies can enhance public health programs for monitor PDDIs and ADRs.

### Limitations

This review is not without limitations. We limited our focus on English apps available in Canada, but other researchers may benefit from extending this review to other regions and languages. Moreover, mHealth apps are frequently updated, and new apps are published daily. Fast evolving app market can limit the generalizability of the results. Another limitation of reviewing app stores is the app databases’ nontransparent search algorithms. Although we reported our search strategy as transparent as possible, the underlying search algorithm can change without the public’s knowledge. This can undermine the reproducibility of our study. Finally, our review unveiled the quality of existing PDDI mHealth apps on the market, but this does not necessarily translate to how consumers use these apps in the real world. This knowledge gap should be further investigated in future research.

### Conclusions

Checking for PDDIs has been a task reserved for clinicians and pharmacists. With the increased popularity of smartphones and other information technologies, they promise more features and functionalities to enhance our lives and well-being. In this study, we searched the most popular mobile app databases and found 23 apps that can check for PDDIs. Some of these apps provided high quality, accurate, and comprehensive information about PDDIs. However, not all apps conformed to high standards, and given the high stake of incorrect drug information, the need for oversight was clear to ensure end-user safety. We also identified secondary features that future apps should incorporate to further benefit the end users. These features can support medication management, improve data security and privacy, and facilitate communications.

## Appendix

Multimedia Appendix 1 Extracted secondary features and their presence among included apps.

Multimedia Appendix 2 A detailed MARS score for all included apps.

Multimedia Appendix 3 List of drug-drug interactions tested for the MARS #15 and #16.

Multimedia Appendix 4 Detailed inter-rater reliability as analyzed by the weighted kappa, intraclass correlation, and Krippendorff alpha for each MARS dimension.

## Abbreviations

ADR: adverse drug reaction
DDI: drug-drug interaction
HCP: health care professional
ICC: intraclass correlation
iOS: iPhone operating system
IQR: interquartile range
IRR: interrater reliability
MARS: Mobile App Rating Scale
mHealth: mobile health
PDDI: potential drug-drug interactions
