# Supplementary material for: Consumer Mobile Apps for Potential Drug-Drug Interaction Check: Systematic Review and Content Analysis Using the Mobile App Rating Scale (MARS)
Source: JMIR Mhealth Uhealth. 2018 Mar 28;6(3):e74. doi: 10.2196/mhealth.8613 (PMC5895923; doi:10.2196/mhealth.8613)
Supplement: Multimedia Appendix 1 [file mhealth_v6i3e74_app1.pdf]

Multimedia Appendix 1. Extracted secondary features and their presence among included apps

App #. App Name	CS	DS	GBN*	MD	MR	MTH	MUS	PP	PI	RR	Total
1. Drug center - pediatric oncall	Y	N	N	Y	N	N	Y	Y	N	N	4
2. Drug interactions	Y	N	Y	N	N	N	Y	N	N	N	3
3. DrugChecker - Interactions (Lite)	N	N	N	N	N	N	N	N	N	N	0
4. Drugs.com Medication Guide	Y	N	Y	Y	N	N	Y	Y	Y	N	6
5. GenieMD	Y	N	Y	Y	Y	Y	Y	Y	N	Y	8
6. MyRxPRofile	N	Y	Y	Y	N	Y	Y	Y	N	N	6
7. PharmaGuide	N	N	Y	Y	N	N	N	N	N	N	2
8. Pharmazam	N	Y	Y	N	N	Y	Y	Y	N	N	5
9. Pharmacist Pro - Drug Interactions Checker	Y	N	Y	Y	N	N	N	N	N	N	3
10. Pill sync drug facts interactions identifier	Y	N	Y	Y	N	N	Y	Y	Y	N	6
11. Prescription Checker	Y	N	Y	N	N	N	Y	N	N	N	3
12. ZibdyHealth	N	Y	Y	Y	Y	Y	Y	Y	N	N	7
13. Assist IE - Drug Interactions	N	N	Y	Y	N	N	N	N	N	N	2
14. Assist UK - Drug Interactions	N	N	Y	Y	N	N	N	N	N	N	2
15. CVS Caremark	Y	N	Y	N	N	Y	Y	Y	Y	N	6
16. Drug Center - Pediatric Oncall	Y	N	N	Y	N	N	Y	Y	N	N	4
17. Drug Interactions	Y	N	Y	N	N	N	Y	N	N	N	3
18. Drugs.com Medication Guide1	Y	N	Y	Y	N	N	Y	Y	Y	N	6
19. Epocrates Plus	Y	N	Y	Y	N	N	Y	Y	Y	N	6
20. GenieMD	Y	N	Y	Y	Y	Y	Y	Y	N	Y	8
21. PillSync Drug Facts Identifier	Y	Y	Y	N	N	N	Y	Y	N	N	5
22. Prescription Checker	N	N	Y	Y	N	N	N	N	N	N	2
23. ZibdyHealth	N	Y	Y	Y	Y	Y	Y	Y	N	N	7
Total	14	5	20	16	4	7	17	14	5	2	104

CS: Customer Support
GBN: Generic and/or Brand Name
RR: Refill Reminder
MR: Medication Reminder
MTH: Medication Tracking History

PI: Pill Identifier
DS: Data Sharing or Export
MUS: Multiple User Support
PP: Password Protection
MD: Medication Database access (search by name then given more detail about a drug)
* counts towards a total number when both generic and brand name can be used for search
