# Supplementary material for: Consumer Mobile Apps for Potential Drug-Drug Interaction Check: Systematic Review and Content Analysis Using the Mobile App Rating Scale (MARS)
Source: JMIR Mhealth Uhealth. 2018 Mar 28;6(3):e74. doi: 10.2196/mhealth.8613 (PMC5895923; doi:10.2196/mhealth.8613)
Supplement: Multimedia Appendix 2 [file mhealth_v6i3e74_app2.pdf]

Multimedia Appendix 2. A detailed MARS score for all included apps.

App #. App Name	Platform	1. Entertainment	2. Interest	3. Customization	4. Interactivity	5. Target Group	6. Performance	7. Ease of Use	8. Navigation	9. Gestural Design	10. Layout	11. Graphics	12. Visual Appeal	13. Accuracy	14. Goals*	15. Accuracy	16. Comprehensiveness	17. Visual*	18. Credibility	19. Evidence Base*
**1. Drug center - pediatric oncall**	Apple	1.5	2	1.5	3	4.5	4.5	4.5	4.5	4.5	4	3.5	2.5	4.5	NA	2.5	0	NA	3	NA
**2. Drug interactions**	Apple	1.5	1.5	2.5	2	2.5	3.5	1.5	2	1.5	2	1	1.5	3	NA	4.4	4.8	NA	2.5	NA
**3. DrugChecker - Interactions (Lite)**	Apple	1	1	1	1.5	3	3	4	3	3.5	3.5	2.5	2	4	NA	0	0	NA	1	NA
**4. Drugs.com Medication Guide**	Apple	3.5	4.5	4.5	5	4	4.5	4.5	4	4.5	4	4	4	5	NA	4.4	5.0	NA	2	NA
**5. GenieMD**	Apple	4	4.5	2.5	4.5	4	4.5	4	4.5	4	3.5	4.5	4.5	4.5	NA	4.8	0	NA	2	NA
**6. MyRxPRofile**	Apple	2	2	1.5	3	3.5	3	2	2	3	3	3	3.5	4.5	NA	3.8	0	NA	1	NA
**7. PharmaGuide**	Apple	1.5	2	1.5	1	2.5	4	3.5	3	3.5	2	2	1	1.5	NA	0	0	NA	1	NA
**8. Pharmazam**	Apple	2.5	4	3	3	3	3.5	2.5	1	2	3	3.5	3.5	4.5	NA	4.4	0	NA	3	NA
**9. Pharmacist Pro - Drug Interactions Checker**	Apple	3	3	1.5	1.5	3.5	5	5	5	5	4	3.5	3.5	5	NA	2.3	0	NA	2.5	NA
**10. Pill sync drug facts interactions identifier**	Apple	2.5	3	2	4	3.5	3.5	3.5	3.5	3.5	3	4	2.5	4.5	NA	4.2	0	NA	1.5	NA
**11. Prescription Checker**	Apple	1.5	1.5	2.5	2	2.5	3.5	1.5	2	1.5	2	1	1.5	3	NA	4.4	4.8	NA	2.5	NA
**12. ZibdyHealth**	Apple	2	2	2	2	2.5	3.5	2	2.5	2.5	2.5	2.5	2	4	NA	0	0	NA	2	NA
**13. Assist IE - Drug Interactions**	Google	2	2.5	1.5	1.5	3	4	5	4.5	5	4	2.5	2.5	4.5	NA	2.3	2.5	NA	2.5	NA
**14. Assist UK - Drug Interactions**	Google	2	2.5	1.5	1.5	3	4	5	4.5	5	4	2.5	2.5	4.5	NA	2.7	2.9	NA	2.5	NA
**15. CVS Caremark**	Google	4	4	2	3.5	4.5	4	4	4	4.5	4.5	4	4.5	5	NA	1.0	3.7	NA	3	NA
**16. Drug Center - Pediatric Oncall**	Google	2.5	3	2	3	4	4.5	4.5	4.5	5	4	3.5	2.5	4.5	NA	2.5	2.7	NA	3	NA
**17. Drug Interactions**	Google	1.5	1.5	2.5	2	2.5	3.5	1.5	2	1.5	2	1	1.5	3	NA	4.4	4.8	NA	2.5	NA
**18. Drugs.com Medication Guide1**	Google	3.5	4.5	4.5	5	4	4.5	4.5	4	4.5	4	4	4	5	NA	4.4	5.0	NA	2	NA
**19. Epocrates Plus**	Google	4	4.5	4	5	4.5	5	4.5	4.5	4.5	4.5	5	5	3	NA	4.0	5.0	NA	3	NA
**20. GenieMD**	Google	4	4	3	4	4	5	3	2.5	3.5	4	4	3.5	5	NA	4.8	0	NA	3	NA
**21. PillSync Drug Facts Identifier**	Google	2.5	3	2	4	3.5	3.5	3.5	3.5	3.5	3	4	2.5	4.5	NA	0	0	NA	1.5	NA
**22. Prescription Checker**	Google	1.5	1.5	2.5	2	2.5	3.5	1.5	2	1.5	2	1	1.5	3	NA	4.4	4.8	NA	2.5	NA

**23. ZibdyHealth**	Google	2	2	2	2	2.5	3.5	2	2.5	2.5	2.5	2.5	2	4	NA	0.8	4.0	NA	2	NA

* NA is a valid option for an answer for MARS
