# Supplementary material for: Consumer Mobile Apps for Potential Drug-Drug Interaction Check: Systematic Review and Content Analysis Using the Mobile App Rating Scale (MARS)
Source: JMIR Mhealth Uhealth. 2018 Mar 28;6(3):e74. doi: 10.2196/mhealth.8613 (PMC5895923; doi:10.2196/mhealth.8613)
Supplement: Multimedia Appendix 3 [file mhealth_v6i3e74_app3.pdf]

Multimedia Appendix 3. List of drug-drug interactions tested for the MARS #15 and #16.

**Drug-Drug Combinations**
Dexmethylphenisate+ Isocarboxazid
Atazanavir+ Omeprazole
Fluoxetine+ Tranylcypromine
Digoxin+ Itraconazole
Irinotecan+ Ritonavir
Irinotecan+ Clarithromycin
Irinotecan+ Ketocanazole
Meperidine+ Tranylcypromine
Tricyclic Antidepressants (TCAs)+ Tranylcypromine
Amiodarone-Procainamide
Remelteon+ Fluvxamine
Bosentan+ Ritonavir
Simvastain+ Amiodarone
Simvastain+ Clarithromycin
Simvastain+ Itraconazole
Indinavir+ Ergotamine
Clarithromycin+ Ergotamine
Ketoconazole+ Ergotamine
Tizanidine+ Ciprofloxacin
Sumatriptan+ Tranylcypromine
**Non- Interacting Pairs**
Acetaminophen/ Codeine+ amoxicillin
Carbamazepine+ Erythromycin Ophthalmic
Metformin+ Erythromycin Opthalmic
Digoxin+ sildenafil
Warfarin+ Digoxin
Warfarin+ Pravastatin
