# Supplementary material for: Consumer Mobile Apps for Potential Drug-Drug Interaction Check: Systematic Review and Content Analysis Using the Mobile App Rating Scale (MARS)
Source: JMIR Mhealth Uhealth. 2018 Mar 28;6(3):e74. doi: 10.2196/mhealth.8613 (PMC5895923; doi:10.2196/mhealth.8613)
Supplement: Multimedia Appendix 4 [file mhealth_v6i3e74_app4.pdf]

Multimedia Appendix 4. Detailed inter-rater reliability as analyzed by the weighted kappa, intra-class correlation, and Krippendorff alpha for each MARS dimension

Table S1: Inter-rater reliability as assessed with the Weighted Kappa (Cohen's)

MARS Dimensions	Kappa	Lower 95% Confidence Interval	Upper 95% Confidence Interval
Engagement	0.59	0.32	0.85
Functionality	0.84	0.75	0.93
Aesthetics	0.84	0.70	0.97
Information	0.96	0.92	0.99

Table S2: Inter-rater reliability as assessed with the Intra-Class Correlation

MARS Dimensions	ICC Coefficient	Lower 95% Confidence Interval	Upper 95% Confidence Interval
Engagement	0.65	0.34	0.83
Functionality	0.83	0.64	0.92
Aesthetics	0.84	0.67	0.93
Information	0.96	0.90	0.98

Table S3: Inter-rater reliability as assessed with the Krippendorff alpha

MARS Dimensions	ICC Coefficient	Lower 95% Confidence Interval	Upper 95% Confidence Interval
Engagement	0.60	0.39	0.76
Functionality	0.84	0.76	0.88
Aesthetics	0.77	0.65	0.86
Information	0.95	0.84	0.97
